# Health-Related Quality of Life and Primi-Gravid: A Comparative Study of Natural Conception and Conception by Assisted Reproduction Technologies (ARTs)

**Published:** 2014-07-08

**Authors:** Seyed Ebrahim Ahmadi, Ali Montazeri, Ramin Mozafari, Afsaneh Azari, Mohammad Reza Nateghi, Mahnaz Ashrafi

**Affiliations:** 1 Iranian Biological Resource Center, Academic Center for Education, Culture and Research (ACECR), Tehran, Iran; 2 Department of Social Welfare, The University of Social Welfare and Rehabilitation Sciences, Tehran, Iran; 3 Mental Health Research Group, Mother and Child Health Research Center, Iranian Institute for Health Sciences Research, ACECR, Tehran, Iran; 4 Child Health Research Center, Tehran Medical Sciences Branch of ACECR, Tehran, Iran; 5 Department of Endocrinology and Female Infertility at Reproductive Biomedicine Research Center, Royan Institute for Reproductive Biomedicine, ACECR, Tehran, Iran

**Keywords:** Conception, Assisted Reproduction Technologies (ARTs), Primigravidity, Quality of Life

## Abstract

**Background:**

Childbearing for the first time is a unique experience. Quality of life is an important indicator in health studies. This study aimed to assess the quality of life of women who were conceived by ARTs and had successful childbirth for the first time and to compare it with quality of life in women who become pregnant naturally and similarly had successful childbirth for the first time.

**Materials and Methods:**

This was a cross sectional comparative study. The accessible sam- ple was recruited from patients attending an infertility clinic and two obstetric and gynecology clinics in Tehran, Iran, during March 2010 to March 2011. In all 276 patients were approached. Of these, 162 women (76 women in natural conception group and 86 women in assisted reproduction technologies group) who met the inclusion criteria were entered into the study. Quality of life was assessed using the 36-item Short Form Health Survey (SF-36). Women completed the questionnaire at two time points: i. last trimester and ii. first month after delivery. Comparison was made between two groups using Mann-Whitney U test and paired samples t test.

**Results:**

Comparing the SF-36 scores between women in natural conception group and ARTs group before childbirth, it was found that natural group had better condition on physical functioning, role limitation due to physical problems, bodily pain and social functioning, while the ARTs group reported better status on general health, vitality, role limitation due to emotional problems, and mental health. However, after childbirth, the ARTs group reported a better condition almost on all measures, except for physical functioning. Comparing differences in obtained scores between two groups before and after childbirth, the results showed that improvements in health related quality of life measures for the ARTs group were greater in all measures, expect for general health.

**Conclusion:**

The findings from this study suggest that health-related quality of life was improved in women who became a mother for the first time by either method. Comparing to women who became mother by natural conception, women who received ARTs showed better quality of life from this first successful experience.

## Introduction

First pregnancy is a major event in woman’s life. Changes in physical appearance, feelings related to motherhood, and family expectations are among challenges that might primigravida women experience more often. Thus, studying health-related quality of life in this population would be worthwhile either when pregnancy occurs naturally or when happens by assisted reproduction technologies (ARTs).

In addition of computation of morbidity indicators, incidence and prevalence rate of diseases and fertility rates, for health assessment and evaluating of the health involvement presently another factor added to the indices of health, is the quality of life (1, 2). Quality of life is an important indicator in health studies in order to be considered as fundamental information and to be ascribed at the time of evaluating interventions (2, 3).

There is a number of studies assessing quality of life of female population indifferent parts of world in related to fertility (4-10) or to different methods of delivery (11-15); however, studies evaluating health-related quality of life among infertile women who became pregnant by ARTs for the first time and had successful childbirth are scarce. In general, the findings of different literature suggest that health-related quality of life in infertile women is suboptimal (15-19), while they might suffer from a poor psychosocial health (20-25), and with regard to the type of delivery, there is no evidence of a clear-cut finding in favor of a given mode of childbirth (11-15).

Child-bearing is one of the most painful procedures that a female is likely to experience. The multi-dimensional feature which is defined to exceed the extreme illness situation (26). Primigravida women expect her first childbearing experience to be frightening, extremely difficult, too long and painful (27).

The objective of this study is twofold. Firstly, we assessed the health-related quality of life scores in women who become pregnant by ARTs and had successful childbirth for the first time, and secondly, we compared obtained scores in health-related quality of life between aforementioned group and women who become pregnant naturally and similarly had successful childbirth for the first time.

## Materials and Methods

### Design and the study samples

This was a cross sectional study of primigravida women in order to compare health-related quality of life between those who had natural conception and those who received ARTs. The women were recruited from the patients attended Royan Institute and two obstetric and gynecology clinics in the north and south of Tehran, Iran, during March 2010 to March 2011. The recruitment of patients from two different settings was due to the fact that we intended to include women with different socio-economic backgrounds in the study.

Accessible samples were selected if they were aged between 20 to 35 years old, experiencing their first conception, living in Tehran and being Iranian. They completed the study questionnaires at two points in time: i. last trimester, and ii. first month after childbirth.

Women with postpartum depression, disability or chronic illness, multifetal, miscarriage, stillbirths, neonatal period death and infant anomaly were excluded from the study. In addition, due to high risk pregnancy for those who became pregnant for the first time at age over 35, we did not include this age group in the study.

Based on results of a study by Nilforooshan et al. (17) and in order to demonstrate a significant difference between two groups regarding quality of life, the sample size was calculated by the following formula, and a sample of at least 65 women per each group was estimated (S=13 and d=10):

$$n=\frac{2{\left(Z_{\left(1\frac{\alpha }{2}\right)}+Z_{1-\beta }\right)}^{2}S^{2}}{d^{2}}$$

A study with such a sample size would have a power of 80% at a 0.05 significance level.

However, in practice, a consecutive sample of 276 patients was approached. Of these, 162 women (76 women in natural conception group and 86 women in assisted reproduction technologies group) met the inclusion criteria, and were then entered into the study and completed questioner before and after childbirth.

### Measures

Health-related quality of life (HRQL) was measured using the Iranian version of 36-item Short Form Health Survey (SF-36). SF-36 was constructed to survey health status by John Ware in 1992 (28), while the Iranian version of the questionnaire underwent a rigor psychometric evaluation by The Institute for Health Sciences Research (IHSR) and showed that it is a reliable and valid measure of health-related quality of life in Iran (29).

The SF-36 measures eight health-related concepts as follows: i. physical functioning (PF-10 items), ii. role limitations due to physical problems (RP-4 items), iii. bodily pain (BP-2 items), iv. general health perceptions (GH-5 items), v. vitality (VT-4 items), vi. social functioning (SF-2 items), vii. role limitations due to emotional problems (RE-3 items), and viii. perceived mental health (MH-5 items). The items can be summed up to give scores from 0 to100 for each subscale. Higher scores show better HRQL (28-31).

"Reliability was estimated using the internal consistency, and validity was assessed using known-groups comparison and convergent validity. In addition factor analysis was performed. The internal consistency (to test reliability) showed that all eight SF-36 scales met the minimum reliability standard, the Cronbach’s alpha coefficients ranging from 0.77 to 0.90 with the exception of the vitality scale (alpha=0.65)" (29).

Demographic and clinical variables including age, education, employment, and previous treatment for infertility were also collected in a separate form.

### Statistical analysis

Descriptive analysis was performed to explore the data. For comparing categorical data, chi-squared test was used, while t test and Mann-Whitney U test were used for group comparisons. The Wilcoxon two related samples test was used to compare the mean values for before and after childbirth assessments. A Statistical Package for the Social Sciences (SPSS; SPSS Inc., Chicago, IL, USA) version 13 was used for data analysis and a P value less than 0.05 was considered significant.

The Ethics Committee of ACECR (Iranian Academic Center for Education, Culture and Research) approved the study. All patients gave oral consent for the study.

## Results

The characteristics of women are shown in table 1. The response rate was about 59% and the mean age for the ARTs group was 29.3 (SD=3.5), while it was 24.2 (SD=3.1) for the natural conception group. Comparing the SF-36 scores between women in natural conception group and ARTs group before childbirth, it was found that natural group had better condition on PF, RP, BP and SF, but the ARTs group reported better status on GH, VT, RE and MH. The Results are shown in table 2. However, after childbirth, the ARTs group reported a better condition almost in all measures, except for physical functioning (Table 3).

**Table 1:** The characteristics of the study sample


	ARTs (n = 86)	Natural conception (n = 76)	
	N (%)	N (%)	P value

**Age**			
Mean (SD)	29.3 (3.5)	24.2 (3.1)	<0.001*
**Education**			<0.001**
Primary	(0) 0	6 (8)	
Secondary	(73) 63	66 (87)	
Higher	23 (27)	4 (5)	
**Employment**			0.524**
Housewife	78 (91)	71 (93)	
Employed	8 (9)	5 (7)	


*; Derived from t test and **; Derived from chi-square test.

**Table 2:** Comparison of SF-36 scores between ARTs and natural conception groups before childbirth using Mann-Whitney U test


	ARTs (n=86)	Natural conception (n=76)	
	Mean (SD)	Mean (SD)	P value

**Physical functioning**	42.4 (28.2)	62.1 (19.3)	< 0.0001
**Role physical**	28.7 (34.6)	41.7 (34.4)	0.018
**Bodily pain**	59.2 (22.5)	64.8 (26.0)	0.148
**General health**	71.1 (19.1)	61.5 (26.0)	0.007
**Vitality**	56.6 (21.4)	54.0 (17.9)	0.394
**Social functioning**	67.9 (23.2)	81.9 (25.6)	<0.0001
**Role emotional **	65.1 (41.2)	58.7 (41.0)	0.328
**Mental health**	71.9 (19.0)	68.5 (18.9)	0.268


**Table 3:** Comparison of SF-36 scores between ARTs and natural conception groups after childbirth using Mann-Whitney U test


	ARTs (n=86)	Natural conception (n=76)	
	Mean (SD)	Mean (SD)	P value

**Physical functioning**	73.5 (23.4)	88.2 (16.6)	<0.0001
**Role physical**	63.9 (42.9)	55.9 (38.2)	0.213
**Bodily pain**	72.9 (24.9)	64.0 (27.9)	0.034
**General health**	76.7 (15.0)	71.8 (22.2)	0.100
**Vitality**	62.3 (17.6)	56.2 (16.5)	0.024
**Social functioning**	82.6 (17.9)	81.8 (23.5)	0.793
**Role emotional **	85.6 (30.9)	59.2 (39.1)	<0.0001
**Mental health**	76.2 (14.4)	69.5 (16.6)	0.006


The comparison within the ARTs group before and after childbirth also indicated significant improvements in all health related quality of life measures, except for mental health. The results are shown in table 4. Similar analysis for the natural conception group was performed and the results are presented in table 5. There were improvements in PF, RP, GH, VT, RE, and MH, expect for BP and SF.

Finally, in comparison of scores between two groups, before and after childbirth, the results showed that improvements in health related quality of life for the ARTs group were greater in all measures, expect for general health. The findings are shown in table 6.

**Table 4:** The comparison of SF-36 score in ARTs group before and after childbirth (n=86) using Wilcoxon two related samples test


	Before	After	
	Mean (SD)	Mean (SD)	P value

**Physical functioning**	42.4 (28.2)	73.5 (23.4)	<0.0001
**Role physical**	28.7 (34.6)	63.9 (42.9)	<0.0001
**Bodily pain**	59.2 (22.5)	72.9 (24.9)	<0.0001
**General health**	71.1 (19.1)	76.7 (15.0)	0.029
**Vitality**	56.6 (21.4)	62.3 (17.6)	0.046
**Social functioning**	67.4 (23.2)	82.6 (17.9)	<0.0001
**Role emotional **	65.1 (41.2)	85.6 (30.9)	0.001
**Mental health**	71.9 (19.0)	76.2 (14.4)	0.085


**Table 5:** Comparison of SF-36 scores in natural conception group before and after childbirth (n=76) using Wilcoxon two related samples test


	Before	After	
	Mean (SD)	Mean (SD)	P value

**Physical functioning**	62.1 (19.3)	88.2 (16.6)	<0.0001
**Role physical**	41.7 (34.4)	55.9 (38.2)	0.008
**Bodily pain**	64.8 (26.0)	64.0 (27.9)	0.836
**General health**	61.5 (26.0)	71.8 (22.2)	0.005
**Vitality**	54.0 (17.9)	56.2 (16.5)	0.302
**Social functioning**	81.9 (25.6)	81.8 (23.5)	0.967
**Role emotional **	58.7 (41.0)	59.2 (39.1)	0.943
**Mental health**	68.5 (18.9)	69.5 (16.6)	0.666


**Table 6:** Comparison of SF-36 mean differences (score after childbirth minus score before childbirth) between ARTs and natural conception groups using Mann-Whitney U test


	ARTs (n=86)	Natural group (n=76)	
	Mean difference (SD)*	Mean difference (SD)	P value

**Physical functioning**	31.1 (34.4)	26.1 (21.2)	0.186
**Role physical**	35.1 (47.8)	14.1 (44.9)	0.004
**Bodily pain**	13.6 (32.6)	-0.82 (34.2)	0.009
**General health**	5.6 (23.3)	10.3 (31.0)	0.407
**Vitality**	5.7 (26.1)	2.2 (18.7)	0.438
**Social functioning**	14.7 (25.5)	-0.16 (33.5)	<0.0001
**Role emotional **	20.5 (55.8)	0.43 (52.6)	0.016
**Mental health**	4.37 (23.2)	0.95 (19.0)	0.281


## Discussion

This study reported on health-related quality of life in a group of women who became mother for the first time either by a natural conception or ARTs. In general, both groups showed improvement in health-related quality of life after successful childbirth.

It seems that the ARTs group reported more benefit from being mother for the first time. Since the ARTs group was not sure whether they would have had a successful childbirth, when the procedure was successful and they became a mother, they indicated a greater improvement. In addition, the successful childbirth for women in the ARTs group might be seen as the end of stigma surrounding infertility. Evidence suggests that infertility is a constant reminder of inability to conceive (32). It is argued that infertility not only impose pressure on women themselves, but also it makes several difficulties for them in the social world including presenting their life story to others and the justification of why they desire to become a mother (33). However, it should be noted that after successful ARTs, quality of life might be improved; otherwise, such interventions might transform infertility from a private pain to a public and prolonged crisis (34, 35).

Also, our own experiences in ARTs clinics suggest that these women receive more support from their partners. In fact, ARTs is the couples’ common efforts to have a child, and thus, these women report better psychological health when they become a mother. It is argued that the good mental health among women and men undergoing ARTs may simply reflect their satisfaction with successful treatment and fulfillment of their hope for parenthood (34). In addition, as noted by Repokari et al. (36), lack of mental health symptoms in women who experience a stressful period of infertility treatment, could be explained by ego defence mobilization, which could be considered as an interesting subject for future study.

The most significant differences observed between two groups before and after childbirth were: role physical, bodily pain, social functioning and role emotional. This finding suggests that some aspects of both physical and mental components were more influenced by the women’s conditions. Perhaps, as discussed earlier, infertile women experience less stress and better global life quality after having a baby than their fertile controls (37).

In order to understand the extent to which the experience of successful childbirth could improve quality of life in infertile women, we compared quality of life in women who received ARTs and those infertile women who became pregnant spontaneously during their life.

## Conclusion

The findings from this study suggest that health-related quality of life improves in women who became a mother for the first time by either ways. After Comparing two groups, our findings revealed that women who receive ARTs might show more benefit from this first successful experience. The results of this study would serve as baseline data to assess the health of pregnant women. Perhaps, this could be investigated in the future studies, while it is noted that the results should be adjusted for confounding variables.

A group of participants in this investigation were infertile women who had become pregnant after infertility treatment, so the results cannot be generalized to infertile women who have not been treated yet. Unfortunately, since the distribution of our data was not normal, we could not adjust the results for age and education, so these findings should be interpreted with caution.

It is suggested to conduct further studies on the mothers’ quality of life in the months following childbirth in order to evaluate breastfeeding and infant feeding conditions.
